# An evaluation of the current patterns and practices of educational supervision in postgraduate medical education in the UK

**DOI:** 10.1007/s40037-016-0280-6

**Published:** 2016-08-03

**Authors:** Priyank Patel

**Affiliations:** Centre for Medical Education, Barts and the London, School of Medicine and Dentistry, London, UK

**Keywords:** Educational supervision, Postgraduate medical education, Trainees, Supervisors, Clinical context, Trainee development, Clinical assessment, E‑portfolio

## Abstract

**Introduction:**

Globally, clinical supervision has been widely adopted and studied. But in the UK, another variant of supervision has developed in the form of educational supervision. The quality of supervision remains highly variable and inadequate time, investment and guidance hinders its ability to actually benefit trainees. Therefore, undertaking a detailed study of the patterns and practices in educational supervision to inform developments in supervisory practice would be extremely beneficial.

**Methods:**

In this mixed methods study, educational supervisors and trainees working within a large London Trust were surveyed online about their experiences of educational supervision. In addition, observations of supervision sessions with a small group of supervisor and trainee pairs followed-up by semi-structured interviews were conducted. The quantitative data were analyzed using statistical software via descriptive statistics. The qualitative data underwent thematic framework analysis.

**Results:**

Both the qualitative and quantitative data revealed that whilst most junior doctors and supervisors value the ideal of educational supervision as a process for engaging in mentoring dialogues, it can become a tick box exercise, devaluing its usefulness and purpose. Trainees highlighted the need for more frequent formal meeting along with better preparation by supervisors. Supervisors would appreciate more support from trusts to help them enhance supervision for trainees.

**Conclusion:**

The effectiveness of educational supervision can be improved with trainees and supervisors engaging in meaningful preparation and proactive communication before meetings. During these formal meetings, improving the quality of feedback and ensuring that regular mentoring dialogues occurred would be highly valuable.

## What this paper adds

- **What is the problem?** Educational supervision is an important aspect of postgraduate medical education in the UK, but remains highly variable in practice.
- **What was the gap in the literature?** It is an under researched aspect of clinical teaching, with no studies having conducted direct observations of educational supervision sessions.
- **What are the theoretical and/or practical implications of this study?** This study provides a framework for enhancing the effectiveness of supervision for both supervisors and trainees to implement into practice. This involves improving communication, preparation and the quality of feedback in formal supervision meetings.

## Introduction

### Formalizing supervision

The essence of supervision in medical education has existed almost as long as the profession itself. The roots of supervision originate from the apprenticeship model, whereby the ‘master’ passes on his or her knowledge and skill to the ‘apprentice’ [1]. Internationally, clinical supervision has been widely adopted and researched [2–4]. But within the UK, over the last 20 years, another variant of supervision has developed in the form of educational supervision [5]. This encompasses supervision of newly graduated foundation doctors and core medical trainees (junior postgraduate residents) to specialist registrar doctors (senior postgraduate residents) by consultant supervisors.

The advent of educational supervision was part of the restructuring of postgraduate medical education from the 1990s onward to ensure that junior doctors could progress through training grades and be guided through more clearly defined career paths [6]. Educational supervision was developed as a form distinct from clinical supervision to ensure that junior doctors received high quality overarching supervision for their education and career guidance [7].

Formalizing the practice of supervision and training supervisors, through the implementation of Calman’s reforms [8] and Modernizing Medical Careers (MMC) in 2005, were considered to be crucial strides to help fulfil an underlying ambition of this process, to provide a genuine benefit to trainees [9]. Educational and clinical supervision have now become formalized components of postgraduate medical education, supporting the process that facilitates a trainee’s progression throughout their training.

From their study, Kilminster and Jolly [10, p. 828] generated a definition of educational supervision as being ‘the provision of guidance and feedback on matters of personal, professional and educational development in the context of a trainee’s experience of providing safe and appropriate patient care.’ This is distinguished from clinical supervision, defined to be ‘an exchange between practising professionals to enable the development of professional skills’ [11, p. 12]. Ideally, these two roles should be separate but in current clinical practice they often appear merged together [5].

### Effectiveness of supervision

The quality of the relationship between the named supervisor and trainee is the single most important factor determining the effectiveness of supervision [12], with the continuity of this relationship being an essential aspect. This relationship needs to be an active partnership between the supervisor and trainee; both are involved in planning and directing. By creating a trusting environment for the trainee to learn in [13], the value of this relationship can be enhanced and supervision is more effective.

Following their national survey of supervisory practice of specialist registrars, Kilminster et al. [12] devised a framework proposing that effective supervision requires direct observation, structured and regular timetabled sessions, reflection and constructive feedback. Trainees also perceive the provision of constructive feedback to be of central importance, as shown by various studies [10, 14, 15]. The General Medical Council (GMC) [16] states how trainees should receive devoted teaching time and specific feedback. Yet it remains questionable whether this advice is reflected in actual practice, as shown by the recent core medical trainee survey [15, p. 153], where trainees felt supervision was limited with nearly half of the ‘meetings lasting just 10–20 minutes’.

There are differences in how supervisors and supervisees evaluate current practice. Educational supervision is deemed effective when supervisors support the progression of their trainees’ learning by providing access to appropriate training opportunities to gain the required proficiencies [5]. Paediatric trainees consistently rate this educational impact of supervision higher than the supervisors, shown by the mixed methods study conducted by Van den Boom et al. [17], a methodological approach rarely employed in supervisory research. Using structured observations and a survey, this study’s conclusion was of the importance of consultants being aware of how trainees valued their input, since consultants often underestimate the significance of supervision to trainees.

Perhaps one of the reasons why supervisors struggle to provide everything trainees want is that those qualified as educational supervisors find themselves with minimal time allocation within their job plans for this role, making it an add-on to their current clinical commitments [12]. The role of named educational supervisor generally equates to an average of 0.25 programmed activities per week for each trainee [18] and is checked as part of the regular quality and contract monitoring by the GMC [19].

Lloyd and Becker [14] acknowledge that both trainees and consultants often consider educational supervision to be an undesirable and administrative obligation rather than a valuable process. This rigid view is further shared by trainees and educational supervisors when using the e‑Portfolio, which they criticized for its inflexibility and lack of specificity [20]. Moreover, educational supervisors devaluing the supervision of foundation doctors by specifically lacking an understanding of the programme [21] further hinders these trainees in truly benefitting from this formal process.

### Problems of educational supervision

Most studies recognize the limited investment in this area and the lack of direction from Royal Colleges about the quality, quantity and structure of supervision further compounds the issue [15, 22]. Whilst the ‘Gold Guide’ for postgraduate specialty training in the UK recommends that educational supervisors should be trained in understanding educational philosophies and practical educational methods, regrettably evidence indicates that this is not the case [9].

Educational supervision forms a crucial component of postgraduate medical education specifically within the UK. Even after formalizing supervisory practice, the presence of many underlying issues such as inadequate time, training, investment and guidance hinders its ability to actually benefit trainees. Whilst clinical supervision has been extensively written about [23], not much currently exists on educational supervision.

The overall purpose of this study was to evaluate the current patterns and practices of educational supervision through a mixed methods approach. In doing so, the following aims can be addressed:

1. Determining the value of educational supervision to both trainees and supervisors.
2. Exploring the process and focus of educational supervision.
3. Identifying ways in which the process of educational supervision can be improved to make it more effective.

This article will specifically report on results pertaining to the third aim of the study.

## Methods

### Research design

A case study research design [24] was the framework used in this small-scale project in evaluating current supervisory practice. The complexity of interpersonal exchange, the defining feature of educational supervision, justified the use of this research design. In this study, educational supervision within one large London trust was the representative case [25], capturing the current situation of a process which occurs in a broader context throughout trusts across England. Being a ‘bounded system’, case study research design is therefore an appropriate technique [26, p. 73].

**Fig. 1:**
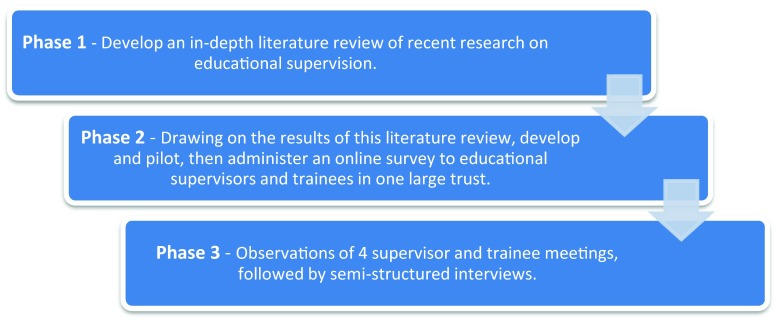
Flow chart illustrating study procedures

This case study used a mixed methods research strategy [27]. It enabled the current patterns and practices of educational supervision to be evaluated quantitatively but also qualitatively, through exploring in-depth the views of both supervisors and trainees based on their experiences of this process. The combination of an online survey, observations and semi-structured interviews allowed triangulation of findings, which ensured greater validity [28]. The procedures of this study are shown in Fig. 1.

### Recruitment

The survey web-link was emailed on 11 February 2015 to the defined inclusion criteria chosen, comprising 462 educational supervisors and 1,125 junior doctors, Foundation Year 1 doctors (newly graduated) upwards, who were being educationally supervised; all were working within the same large London trust. The survey was live for 26 days. During this time two reminder emails were sent to counteract the low response rate associated with survey fatigue [29]. Four supervisor and trainee pairs were also recruited via convenience sampling, to conduct narrative observations and semi-structured interviews using a flexible question prompt.

Two complementary online surveys were designed for supervisors and trainees. These were initially piloted with a few trainees and supervisors accessed through convenience sampling, maximizing validity and generalizability. This culminated in a fully-designed online survey on REDCap (Research Electronic Data Capture), a secure web-based application for constructing and managing online surveys [30].

Ethical approval was obtained from the Quality Improvement Department for Barts Health NHS Trust and Queen Mary Research Ethics Committee. Informed consent for participation was acquired. Anonymity and confidentiality was preserved.

### Data analysis

Quantitative data from the online surveys on REDCap were exported into IBM SPSS Version 22 (Statistical Analysis Software Package). Using this statistical software, the data were tabulated and analyzed via descriptive statistics. Qualitative data, even from the online surveys, underwent thematic analysis using Ritchie and Spencer’s [31] Framework Method, an inductive five-step approach.

All interviews and observations were conducted and transcribed verbatim by the two researchers. From reading the transcripts, the initial coding framework was developed using the emerging themes from the data. Sections of the data corresponding to a specific theme were then indexed and placed in charts consisting of headings and subheadings. This formed the thematic framework, enabling subsequent mapping and interpretation [31]. A quality check of the coding framework was performed by the two researchers to maximize reliability, ensuring rigour in the analysis.

## Results

The quantitative data emerge from the two online surveys, for educational supervisors and trainees respectively. The qualitative data originate from the narrative observations of supervision meetings, semi-structured interviews and an open box question from the online surveys asking both trainees and supervisors about how to improve supervision to make it more effective.

### Online surveys – response rates and demographics

The response rate for both online surveys was low, as shown in Table 1. Only 10.4 % (*n* = 117) of trainees completed the ‘Junior Doctor Version’ compared with 34.6 % (*n* = 160) of supervisors who completed the ‘Supervisor Version’ of the online surveys. Most trainees and supervisors were working in medicine. The majority of the trainees who participated in the survey were foundation doctors.

**Tab. 1:** Demographics, specialty and level of training of trainees and educational supervisors who completed the online surveys

Demographics	Trainees	Supervisors	Level of training	Trainees in %
No. of respondents	117	160	FY1–FY2	43.6
Response rate	10.4 %	34.6 %	CT1–CT2	22.2
Gender–Male	39 %	56 %	ST3–ST4	12.0
Gender–Female	61 %	44 %	ST5–ST6	9.4
			ST7–ST8	6.8
			Other	6.0

Any quotations used are labelled according to which interview transcript (INTRV) or observation narrative (OBS) they are from, along with its page number. Quotations used from the open box questions of the online surveys are labelled as survey response (SR).

### Value of educational supervision

Online survey responses showed how 38 % (*n* = 44) of trainees reported that consultant supervisors would rate this process as being highly or extremely important. This contrasts the 93 % (*n* = 149) of supervisors who stated that this process is somewhat or very valuable to trainees. It seems that supervisors know how valuable the process of educational supervision is to trainees, but this value is not being shown clearly enough to their trainees.

**Fig. 2:**
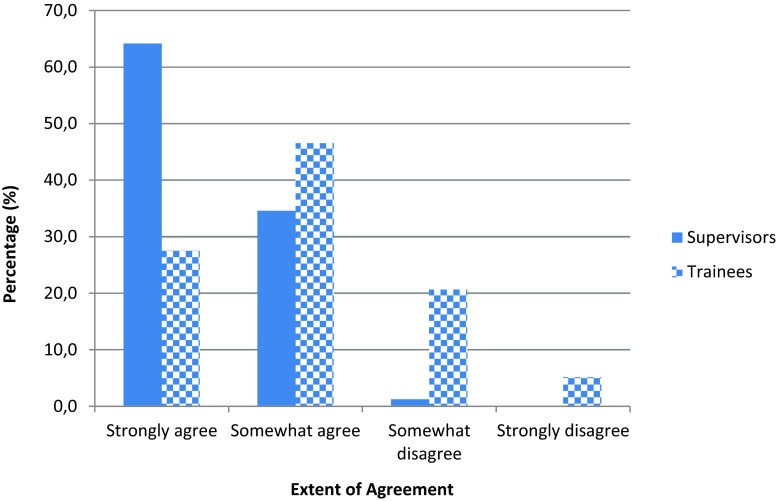
Educational supervision provided is tailored to the level of trainee’s experience and training

Similarly, when considering the value they place upon each other, virtually all supervisors feel somewhat or very valued by their trainees, seen in Fig. 2. In contrast, only 73 % (*n* = 85) of trainees feel somewhat or very valued by their current supervisor. Trainees expressed in the free text responses that they would feel more valued if supervisors *‘show some interest’* by *‘tailoring the meeting around their needs’* and to *‘not view this process (of supervision) as a tick box exercise’* (SR).

Educational supervision is more valued when it is tailored according to the trainee’s level of training, as *‘you get a more personalized review’* (INTRV 2, p. 1). Nearly all supervisors (98.7 %, *n* = 158) somewhat or strongly agree that current supervisory practice is tailored to the appropriate stage of training. In contrast, this view was expressed by just 74.1 % (*n* = 87) of trainees, as shown by Fig. 2. Recognizing that 44 % (*n* = 51) of trainees who completed the online survey were foundation doctors (junior trainee doctors) may suggest that their supervision is not personalized enough. This viewpoint was confirmed by a specialist registrar (senior trainee doctor), who from experience clarified that educational supervisors are *‘more invested in you as a registrar’* (INTRV 3, p. 1).

Educational supervisors benefit from being valued and supported by their respective trusts (organizations providing healthcare to designated geographical areas), which appoint and regularly appraise their practice. Interestingly, 62.5 % (*n* = 100) of supervisors feel somewhat undervalued or not valued by their trust despite the fact that 93.1 % (*n* = 149) of supervisors find their role somewhat or very fulfilling to undertake.

### Process of educational supervision

#### Communication

For supervision to occur, contact needs to be made to arrange a time and place. There appears to be a difference in opinion between supervisees and supervisors. Specifically, 88.9 % (*n* = 104) of trainees stated they initiated contact with their supervisor, whilst 45.6 % (*n* = 73) of supervisors stated they made the initial contact to their trainee. Contact is usually initiated to arrange a meeting for a formal supervision session, but the results suggested that communication should involve more than that. Supervisors would prefer if *‘objectives were shared before the session’* in order to *‘complete as much as possible prior to meeting’* (SR), to make sessions more effective. Similarly, trainees felt that *‘minimal email contact’* (SR) by their supervisor hindered communication.

#### Duration and number of meetings

**Fig. 3:**
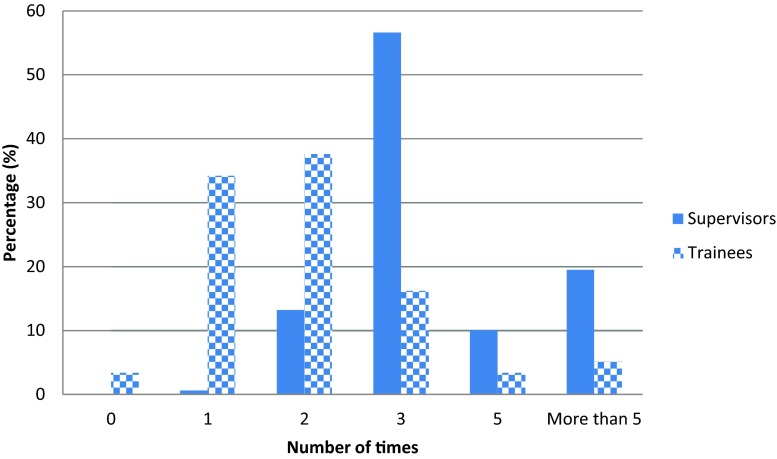
The number of times formal supervision sessions occurred according to trainees and supervisors

When considering the number of times supervisors and trainees actually met, 16.2 % (*n* = 19) of trainees and 56.3 % (*n* = 90) of supervisors said that three formal meetings occurred over the course of a training post. Furthermore, 29.5 % (*n* = 47) of supervisors said they met five times or more, yet only 8.5 % (*n* = 10) of trainees stated this. Hence, trainees expressed the need for more *‘formal frequent supervision meetings’* (SR) as the vast majority of trainees had just one or two formal meetings during a rotation, as seen in Fig. 3.

### Improving the effectiveness of educational supervision

**Fig. 4:**
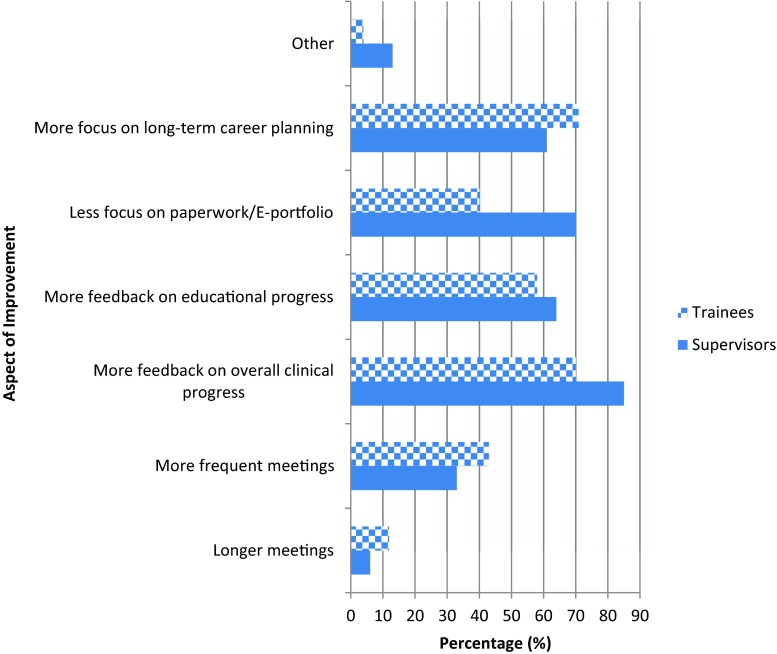
Aspects of educational supervision that need to be improved to make the process more effective based on trainees and supervisors experience of supervision to date

Most of the results related to this theme originated from the online surveys. Fig. 4 highlights the results from a multiple choice question on aspects of supervision that could be improved to make the process more effective. Both supervisors and trainees were asked to check up to three areas most important to them. In addition to this, the open box question, which was dedicated to exploring this theme, provided more in-depth responses.

#### Feedback

Obtaining more feedback on clinical and educational progress were the two most important aspects that, if improved, would make supervision more effective according to both trainees and supervisors, illustrated by Fig. 4. Free text responses further supported this, as trainees ideally wanted *‘constructive feedback against current curricula and training needs’* along with *‘specific examples’* on how to improve (SR). This may be achievable if the focus on paperwork/e-Portfolio is reduced, an aspect which supervisors feel slightly more strongly about than trainees, depicted in Fig. 4.

#### Preparation

Reading the e‑Portfolio was the most common method of preparation for educational supervision. However, a number of supervisors felt this was insufficient for trainees. Supervisors want their trainees to have *‘specific questions/goals/objectives in mind before the meeting’* (SR). As this enables trainees to value the process and also think about how exactly their supervisor can best help in achieving their goals.

Likewise, supervisors need to be aware of the trainee’s curriculum in order to provide feedback against the curriculum objectives. One supervisor acknowledged the difficulty in keeping up with curriculum requirements *‘as they change so much’* (INTRV 4, p. 1). But as one supervisor pointed out, undertaking the role of an educational supervisor *‘requires investment’ *and therefore *‘needs to be genuine input’* (INTRV 1, p. 1).

#### Pastoral guidance (mentoring role)

Trainees and supervisors stressed the significance of pastoral support, the notion of helping address personal needs and problems, within their free text responses in making educational supervision more effective. Numerous supervisors want trainees to be *‘open about any concerns they may have so that they can be addressed’* (SR). Contrastingly, various trainees would prefer if the supervisor asked about *‘difficulties outside medicine’* and *‘about personal life/circumstances/issues’* (SR).

## Discussion

**Fig. 5:**
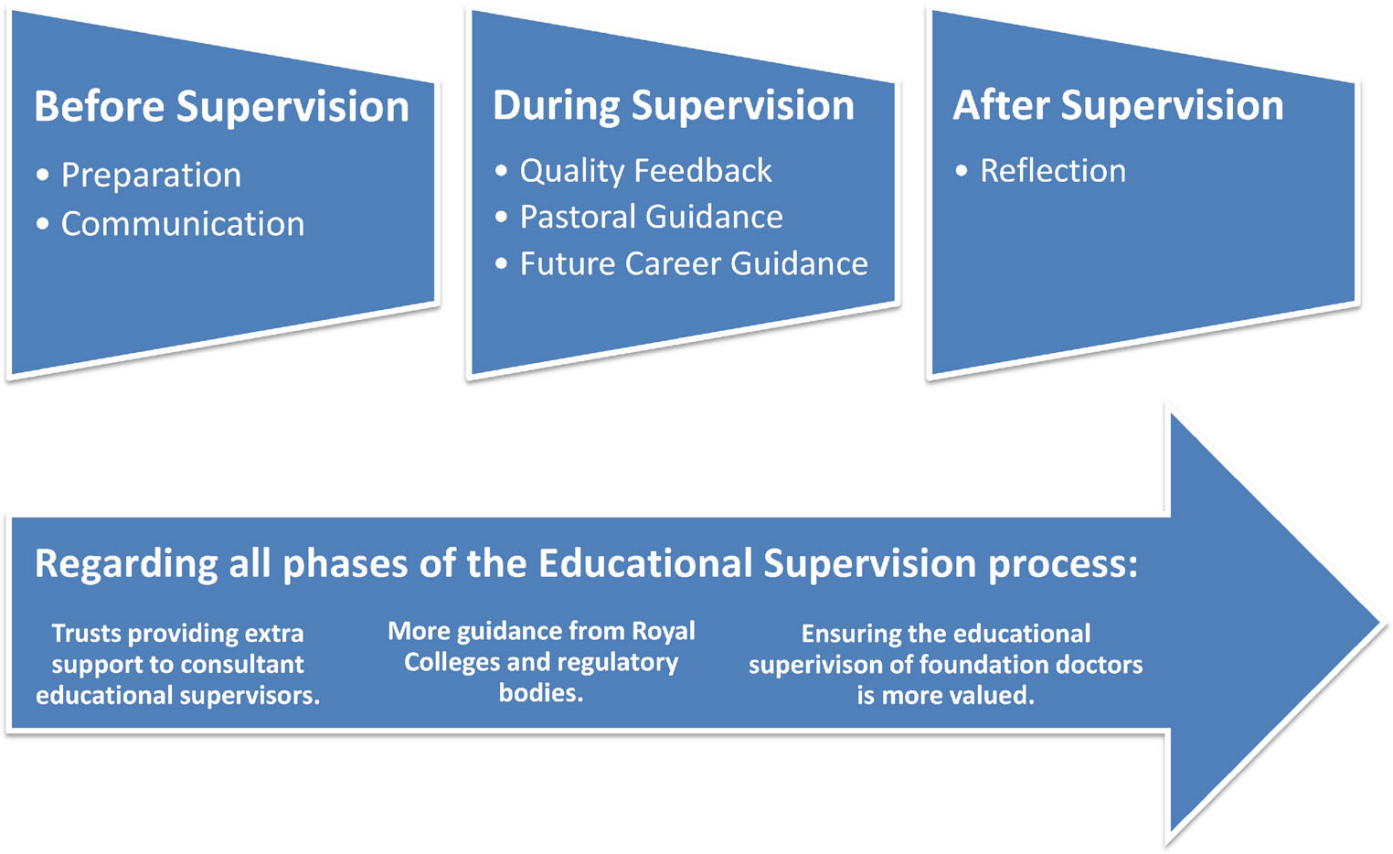
Framework for improving the effectiveness of educational supervision based on findings from this study and is applicable to both trainees and educational supervisors

Based on findings from this study, a framework for improving the effectiveness of educational supervision was developed, shown in Fig. 5. The components of this framework will now be discussed in turn with respect to trainees and supervisors, advising them as to how they can make the process of supervision more meaningful.

### Before supervision – preparation and communication

#### Trainees

The importance of preparation by trainees prior to educational supervision sessions was emphasized by the majority of the supervisors. This study crucially showed how both trainees and supervisors need to be more proactive in their communication to improve the effectiveness of sessions. Trainees’ communicating their ideas and concerns prior to formal sessions is appreciated by supervisors as the online surveys revealed, in line with a finding presented by Murdoch-Eaton et al. [32].

#### Supervisors

Educational supervisors can demonstrate how they value supervision by the extent to which they tailor this process to meet their trainee’s needs. Whilst virtually all supervisors agreed that supervision is tailored, a number of trainees felt that supervisors lacked awareness of their curriculum requirements.

Educational supervisors inadequately preparing and thereby lacking understanding of the Foundation programme in particular has been previously reported by second-year Foundation doctors [21]. This subsequently impacts the quality of feedback, which trainees believe should be delivered according to curriculum objectives.

### During supervision – quality feedback and pastoral guidance

#### Trainees

Discussing the key aspects of feedback during supervision is important to trainees. Findings from this study supported the work by Lloyd and Becker [14], where paediatric specialist registrars recognized constructive feedback, career planning and goal setting as crucial elements of supervision. However in the online survey, free text responses from the trainees suggested that these areas required more emphasis to make supervision meaningful. Moreover, pastoral guidance (i. e. mentoring support) needed to be pursued further by trainees according to supervisors.

The majority of the feedback offered to trainees originated from the e‑Portfolio, as recognized from the observations conducted in this study. Reducing this time spent concentrating on the e‑Portfolio could provide an opportunity for career guidance and mentoring dialogues, which trainees believe is hardly offered by supervisors. Thus, trainees feel the advice and time which supervisors are able to offer can be used more efficiently.

#### Supervisors

Educational supervisors in this study stated they gave more feedback than trainees stated they received, which corresponded with the results from a national survey conducted by Grant et al. [33]. This could be explained by trainees not considering the informal guidance and advice provided by consultant supervisors as feedback, which forms part of the informal curriculum [34]. The value of such informal learning within postgraduate medical education has been previously emphasized by Swanwick [35].

When offering constructive feedback to trainees, whether done formally or informally, supervisors need to be aware of curriculum requirements. This is particularly relevant when focussing on the educational supervision of foundation trainees (newly qualified doctors), which can be somewhat undervalued by supervisors as the results suggest. This may prove controversial as registrars, being more senior, are prioritized as they will become future colleagues sooner.

Supervisors should provide mentoring and guidance to both foundation doctors and registrars equally. Supervisors have to be proactive in ensuring the relationship with their trainees continues to strengthen by engaging in informal meetings prior to commencing a post. This was a fundamental method identified in building rapport from the qualitative branch of this study, ensuring trainees can comfortably raise any anxieties or concerns.

### After supervision – reflection

Discussing reflection during formal supervision meetings was a vital theme identified when conducting observations in this study. Educational supervisors were observed underlining the importance of reflection-on-action to trainees, in line with Schön’s reflective practitioner model [36], a framework enabling professionals to learn from experience. Trainees should further engage in ‘reflection-on-feedback’, with the aim of implementing these approaches into practice [37]. The need for greater discussion of reflection between supervisors and trainees was formerly identified by Kilminster and Jolly [10].

### Limitations of this study

A major limitation of this study was the low response rate to the survey, despite a number of reminder emails being sent. Educational supervisors still outnumbered trainees in terms of response rate by nearly 2:1, even though three times as many trainees were contacted. Nevertheless, the quantitative results offered some useful insight into the current patterns and practices of educational supervision, which were supported by the qualitative findings.

This study needs to be repeated on a larger scale, encompassing trusts across England to provide a national picture of educational supervision. More supervisor and trainee pairs need to be observed and interviewed to strengthen the consistency of findings, made difficult in this study due to time limitations. The themes emerging following analysis of this study’s results can be employed to better enhance the online surveys, addressing key issues that were underexplored. Introduction of these additional variables would improve quantitative analysis, contributing to a more robust triangulation of findings. Furthermore with time, supervisory practice within individual specialties can be thoroughly examined, assisting Royal Colleges to develop their guidance and frameworks.

### Implications and recommendations

The recent increase in the provision of resources available for educational supervision in the UK has rendered this evaluation of the patterns and practices of educational supervision in one large London trust timely and appropriate. This study has provided a vital insight into a better understanding of current educational supervision, considering the scarcity of research on this subject. It is evident that the process of educational supervision is valuable to trainees, despite some supervisors underestimating its significance to junior doctors. Underlining to supervisors the importance of this process to trainees could be helpful in getting supervisors to value it more. Results of this study also show that National Health Service organizations could be more appreciative of the work educational supervisors undertake, since at present the majority of the supervisors feel undervalued.

## Conclusion

This study has indicated a number of ways that the effectiveness of educational supervision, could be improved as illustrated by the framework in Fig. 5. Both trainees and supervisors need to incorporate these aspects into current practice in order to enhance the true value of educational supervision.

Considering how the origin of educational supervision is deeply rooted within the apprenticeship model, one can argue that supervisors should in fact be taking the lead in organizing and directing this process for trainees. In contrast, recent developments in postgraduate medical education underline the significance of a trainee-led curriculum, encouraging trainees to take more responsibility of their learning.

However, further in-depth research over a longer timeframe is still required to obtain a deeper understanding of educational supervision, in order to offer more concrete guidance and address underlying issues.

## References

[1] Lateef F (2010). Simulation-based learning: Just like the real thing. J Emerg Trauma Shock.

[2] Hore CT, Lancashire W, Fassett RG. Clinical supervision by consultants in teaching hospitals. 0025–729X (Print). http://www.ncbi.nlm.nih.gov/pubmed/19705984 10.5694/j.1326-5377.2009.tb02758.x 19705984

[3] Farnan JM, Petty LA, Georgitis E (2012). A systematic review: the effect of clinical supervision on patient and residency education outcomes. Acad Med.

[4] Baldwin DWC, Daugherty SR, Ryan PM (2010). How Residents View Their Clinical Supervision: A Reanalysis of Classic National Survey Data. J Grad Med Educ.

[5] Abdulla A. Educational supervision: a new challenge. J R Soc Med. 1012008. p. 6. 10.1258/jrsm.2007.070342 PMC2235921 18263906

[6] Dillner L (1993). Senior house officers: the lost tribes. BMJ.

[7] Harris E, Ferreira P (1997). Training senior house officers. BMJ.

[8] Department of Health (1993). Hospital doctors: training for the future. Working Group on Specialist Medical Training.

[9] Cooper N, Forrest K (2009). Essential guide to educational supervision in postgraduate medical education.

[10] Kilminster SM, Jolly BC (2000). Effective supervision in clinical practice settings: a literature review. Med Educ.

[11] Butterworth T, Faugier J (1992). Clinical supervision and mentorship in nursing.

[12] Kilminster S, Cottrell D, Grant J, Jolly B (2007). AMEE Guide. Eff Educ Clin Supervision Med Teach.

[13] Taherian K, Shekarchian M (2008). Mentoring for doctors. Do its benefits outweigh its disadvantages?. Med Teach.

[14] Lloyd BW, Becker D (2007). Paediatric specialist registrars’ views of educational supervision and how it can be improved: a questionnaire study. J R Soc Med.

[15] Tasker F, Newbery N, Burr B, Goddard AF (2014). Survey of core medical trainees in the United Kingdom 2013 – inconsistencies in training experience and competing with service demands. Clin Med.

[16] General Medical Council (2001). Good Medical Practice.

[17] van den Boom M, Pinnock R, Weller J, Reed P, Shulruf B (2012). Paediatric trainee supervision: management changes and perceived education value. J Paediatr Child Health.

[18] Deanery L (2012). Educational Tariff Guidance London: London Deanery.

[19] Council GM (2013). Recognition and approval of trainers: General Medical Council.

[20] Beard J, Strachan A, Davies H (2005). Developing an education and assessment framework for the Foundation Programme. Med Educ.

[21] O’Brien M, Brown J, Ryland I (2006). Exploring the views of second-year Foundation Programme doctors and their educational supervisors during a deanery-wide pilot Foundation Programme. Postgrad Med J.

[22] Cottrell D, Kilminster S, Jolly B, Grant J (2002). What is effective supervision and how does it happen? A critical incident study. Med Educ.

[23] Irby DM (1978). Clinical teacher effectiveness in medicin. Acad Med.

[24] Stake RE. The Art of Case Study Research. SAGE Publications; Thousand Oaks, CA 1995.

[25] Flyvbjerg B (2006). Five Misunderstandings About Case-Study Research. Qual Inq.

[26] Creswell JW. Qualitative Inquiry and Research Design: Choosing Among Five Approaches. SAGE Publications; Thousand Oaks, CA 2007.

[27] Bryman A. Mixed Methods. SAGE Publications; London 2006.

[28] Bryman A (2012). Social research methods.

[29] Porter SR, Whitcomb ME, Weitzer WH (2004). Multiple surveys of students and survey fatigue. NDIR.

[30] Harris PA, Taylor R, Thielke R, Payne J, Gonzalez N, Conde JG (2009). Research electronic data capture (REDcap) – A metadata-driven methodology and workflow process for providing translational research informatics support. J Biomed Inform.

[31] Ritchie J, Spencer L, Bryman A, Bryman PSRA, Burgess B (1994). Qualitative Data Analysis for Applied Policy Research. Analyzing Qualitative Data.

[32] Murdoch-Eaton D, Cass H, Cunnington F, editors. Paediatric Educators’ Programme (PEP) – using pre-course portfolios for course design and participant selection 2008.

[33] Grant J, Kilminster S, Jolly B, Cottrell D (2003). Clinical supervision of SpRs: where does it happen, when does it happen and is it effective? Specialist registrars. Med Educ.

[34] Witman Y (2013). What do we transfer in case discussions? The hidden curriculum in medicine. Perspect Med Educ.

[35] Swanwick T (2005). Informal learning in postgraduate medical education: from cognitivism to “culturism”. Med Educ.

[36] Schön DA (1983). The reflective practitioner: how professionals think in action.

[37] Lefroy J, Watling C, Teunissen WP, Brand P (2015). Guidelines: the do’s, don’ts and don’t knows of feedback for clinical education. Perspect Med Educ.

